# Reduction in the Occupation Ratio of the Supraspinatus Muscle on CT Following a Simulated Acute Retracted Rotator Cuff Tear

**DOI:** 10.7759/cureus.83769

**Published:** 2025-05-09

**Authors:** Nicolaas Kotze, David O'Briain, Peter Jemelik

**Affiliations:** 1 Trauma and Orthopedics, University Hospital Waterford, Waterford, IRL; 2 Trauma and Orthopedics, Connolly Hospital Blanchardstown, Dublin, IRL

**Keywords:** goutallier, occupation ratio, reparability of supraspinatus tear, rotator cuff tears, supraspinatus muscle atrophy, supraspinatus tendon tear

## Abstract

Objective

This study was designed to determine the supraspinatus cross-sectional area and occupation ratio in a simulated retracted rotator cuff tear to quantify the reduction of the supraspinatus volume during acute simulated retraction.

Methodology

Eight CT shoulder scans of patients with normal rotator cuff anatomy were analyzed. CT scans were reoriented to create the Y-view in the oblique sagittal plane. The supraspinatus muscle and fossa cross-sectional areas were measured at the Y-view, and the occupation ratio was calculated. The supraspinatus muscle was also measured at 5, 10, 15, and 20 mm increments toward its insertion, to permit simulation of retraction. The volume of the simulated retracted supraspinatus muscle was extrapolated to the fossa size in the Y-view to create simulated occupation ratios at above mentioned increments. The data set was collected by three different surgeons on two occasions.

Results

Four-level model analysis showed a decrease in supraspinatus muscle surface area and thus occupation ratio at 5-, 10-, 15-, and 20-mm intervals (*P* < 0.001).

Conclusions

Rotator cuff tear is a common presentation in elderly populations. The supraspinatus is a fusiform muscle whose cross-sectional diameter decreases towards its insertion. When determining the reparability of supraspinatus tears, the atrophy and fatty degeneration of the muscle are often used. In a simulated acute supraspinatus tear, muscle retraction led to a significant reduction in cross-sectional area when viewed in the Y-view. This could be misdiagnosed as atrophy, and surgeons should consider this when determining the reparability of acute rotator cuff tears.

## Introduction

The potential reparability of rotator cuff tears has been determined by multiple studies, taking into account the clinical and radiographic parameters [1-6]. Preoperative imaging has been used to aid in decision-making to determine muscle atrophy and fatty infiltration of the muscle. Fatty infiltration was originally described on axial CT scans by Goutallier et al. [7], but more recently, MRI scans in the parasagittal view have been used to determine the fatty infiltration (modified Goutallier grade) and muscle bulk (Tangent sign and occupation ratio) [8]. The Y-view has been widely used to determine these predictive factors [9-11].

This study hypothesizes that supraspinatus repairability according to existing classifications has been too nihilistic in acute tears. Retraction of the rotator cuff tendons following a tear has been well described [12-14]. Retraction could lead to false-positive Tangent sign and decreased occupation ratio that can be misconstrued as atrophy, and thus define acute repairable tears as irreparable. It has been proposed that the conventional Y-view might not be adequate to measure muscle bulk, and a separate measurement should be taken at the level of the maximal cross-sectional area [15]. Standard imaging often does not image far enough medially to visualize the maximal diameter of the rotator cuff muscles, limiting the applicability of this approach.

Studies have shown that both the supraspinatus occupation ratio and the Tangent sign decrease in the Y-view when a rotator cuff tear is present [16]. Jo et al. confirmed the improvement of the occupation ratio in the Y-view postoperatively [17]. This was noted on scans three days postop and as such relates to correction of retraction rather than improvement in muscle bulk or fatty infiltration through rehabilitation. Our study used preoperative CT scans on healthy rotator cuffs to determine if a simulated acute retracted rotator cuff tear in a healthy cuff would be considered as potentially irreparable in the Y-view.

## Materials and methods

Shoulder CT scans from patients with intact rotator cuffs and without severe distortion of normal anatomy were analyzed. These scans were obtained from the National Integrated Medical Imaging System (NIMIS) radiology database at the University Teaching Hospital in the Republic of Ireland, treating trauma and elective orthopedic patients between January 1, 2022, and August 31, 2022. The inclusion criteria encompassed native shoulders of both males and females aged 60-75 years, as this is the age group most affected by rotator cuff tears. CT scans were employed as they could be reformatted to provide images of diagnostic quality at the idealized Y-view. MRI scans were typically performed in this age group for cuff pathology and were excluded. In addition, the MRI scans were not routinely taken in the appropriate plane for Y-view assessment and could not be reformatted.

Excluded from the study were patients with any existing rotator cuff pathology, glenohumeral joint deformity, scapular fractures, and displaced humeral head fractures. A total of 116 shoulder CT scans were identified, of which 68 were excluded for falling outside the specified age range. Of the remaining 48 scans, 40 were excluded due to the presence of exclusion criteria pathologies as previously described.

Of the 8 included scans, 4 were performed to asses for occult osteoarthritis, 2 for proximal humeral fractures with minimal displacement that did not affect supraspinatus anatomy, and 2 for identifying possible pathology where none was found. The study cohort comprised 4 males and 4 females, with 6 right shoulder scans and 2 left shoulder scans.

**Figure 1:**
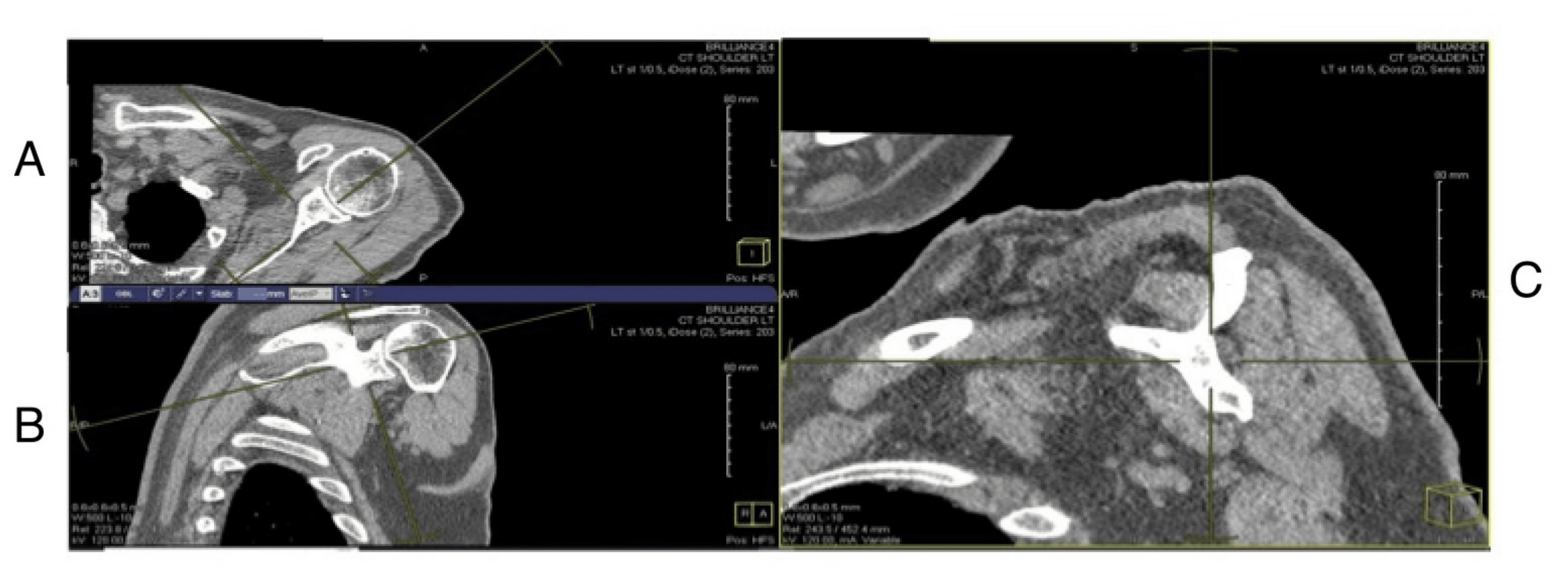
Creating the Y-view of the shoulder on CT images: (A) axial view, (B) coronal view, and (C) oblique sagittal view used to generate the Y-view.

The scans were reoriented using multi-plane reconstruction techniques to recreate the Y-view described by Thomazeau et al. [9]. Briefly, an oblique sagittal view was generated by aligning the coronal plane with the glenoid fossa and the axial plane with the scapular spine. The oblique sagittal view was translated medially until the coracoid process connected with the scapular spine, forming the Y, as shown in Figure 1. Window contrast levels were adjusted to *soft tissue* parameters to optimally visualize the rotator cuff musculature. These images were then used for measurements.

**Figure 2:**
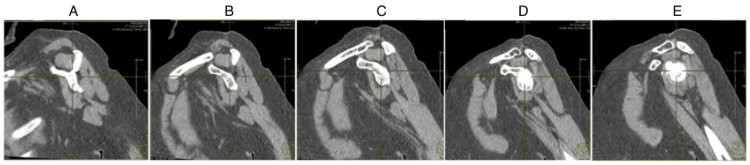
Simulating a retracted supraspinatus tear using the same CT scan and creating a more lateralized view: (A) the Y-view, (B) image moved 5 mm laterally, (C) image moved 10 mm laterally, (D) image moved 15 mm laterally, and (E) image moved 20 mm laterally.

To simulate a tear, images were also produced at 5, 10, 15, and 20 mm lateral to the original plane (Figure 2). These images provided the cross-sectional view of the tendon/muscle as it would appear at the Goutallier point if retracted by 5, 10, 15, or 20 mm, respectively. This process was repeated for all patients. To prevent observer bias, the images were anonymized and the order randomized.

**Figure 3:**
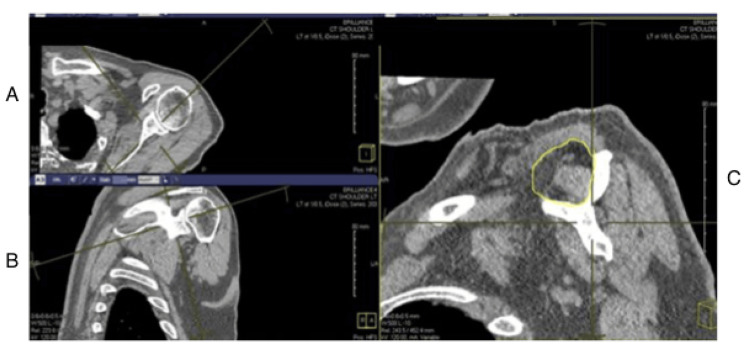
Measuring the supraspinatus fossa on the Y-view: (A) axial view, (B) coronal view, and (C) oblique sagittal view on which the Y-view is created and measurements are made.

Measurements were made using Image J software (National Institutes of Health [NIH], Bethesda, MD) [18]. The supraspinatus fossa area was measured using the scapular spine, coracoid process, and the inferior border of the trapezius muscle as described by Werthel et al. [19]. This measurement can be seen in Figure 3.

**Figure 4:**
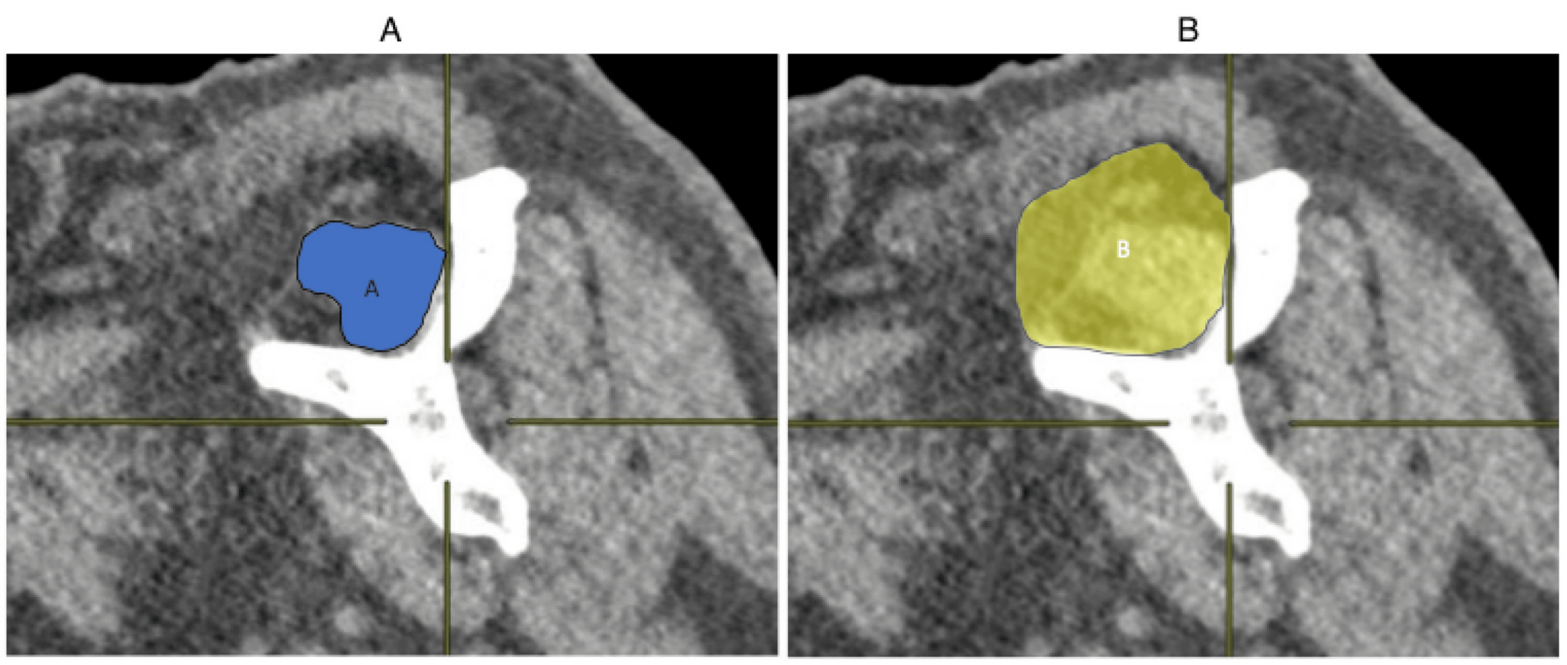
Occupation ratio measured on the Y-view. (A) Supraspinatus muscle surface area measured in the Y-view, (B) supraspinatus fossa surface area measured in the Y-view. Occupation ratio = A/B.

The occupation ratio was calculated by dividing the supraspinatus muscle cross-sectional surface area by the supraspinatus fossa surface area in the Y-view (Figure 4). Inter- and intra-observer reliability were determined by repeating measurements after three months. A total of 18 measurements were obtained per image (3 measurements × 3 observers × 2 occasions).

Statistical methods

The observer-specific (*k* = 3) and replicate-specific (*n* = 2) measurements for supraspinatus surface areas and their corresponding occupation ratios for each patient (*n* = 8) at each distance (0, 5, 10, 15, and 20 mm) were plotted, yielding a total of 240 values. A preliminary analysis confirmed that the within-observer variance was negligible. To evaluate how supraspinatus surface areas varied across simulated tear distances, we used linear mixed-effects models with crossed random effects for both observer and patient.

The Akaike Information Criterion (AIC) was employed in this study to compare two statistical models examining the relationship between simulated tear distances and supraspinatus surface area, adjusted for fossa area at 0 mm. AIC is widely recognized for model selection as it balances goodness of fit with model complexity by considering the number of parameters. By applying AIC, we were able to objectively determine which model, one assuming a linear effect of distance (expressed as mm²/mm) or the other estimating the mean surface area at each distance, better represents the data. Additionally, all estimates are reported with 95% confidence intervals and *P*-values based on Wald standard errors, which further supports clearer and more reliable interpretations of the relationship being studied [20].

## Results

**Figure 5:**
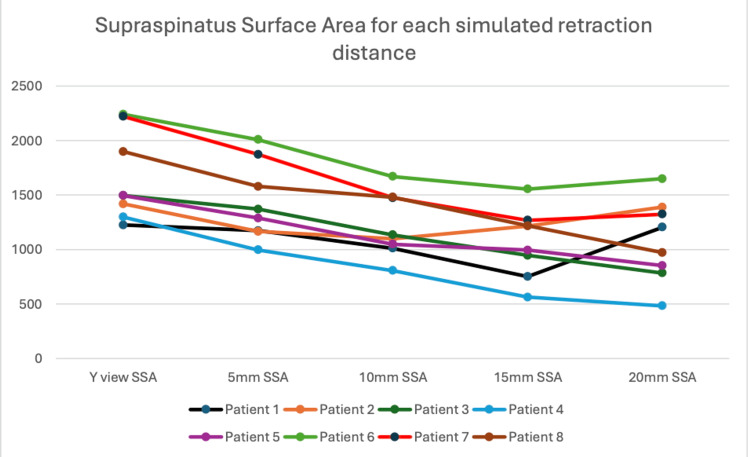
Supraspinatus surface areas by patient at each distance, comparing overall mean values with observer/replicate-specific values. SSA is measured in ImageJ units and does not reflect a metric value. SSA, supraspinatus surface area

Patient- and distance-specific supraspinatus surface areas are displayed in Figure 5. Supraspinatus surface areas decreased with larger simulated tear distances. 

**Figure 6:**
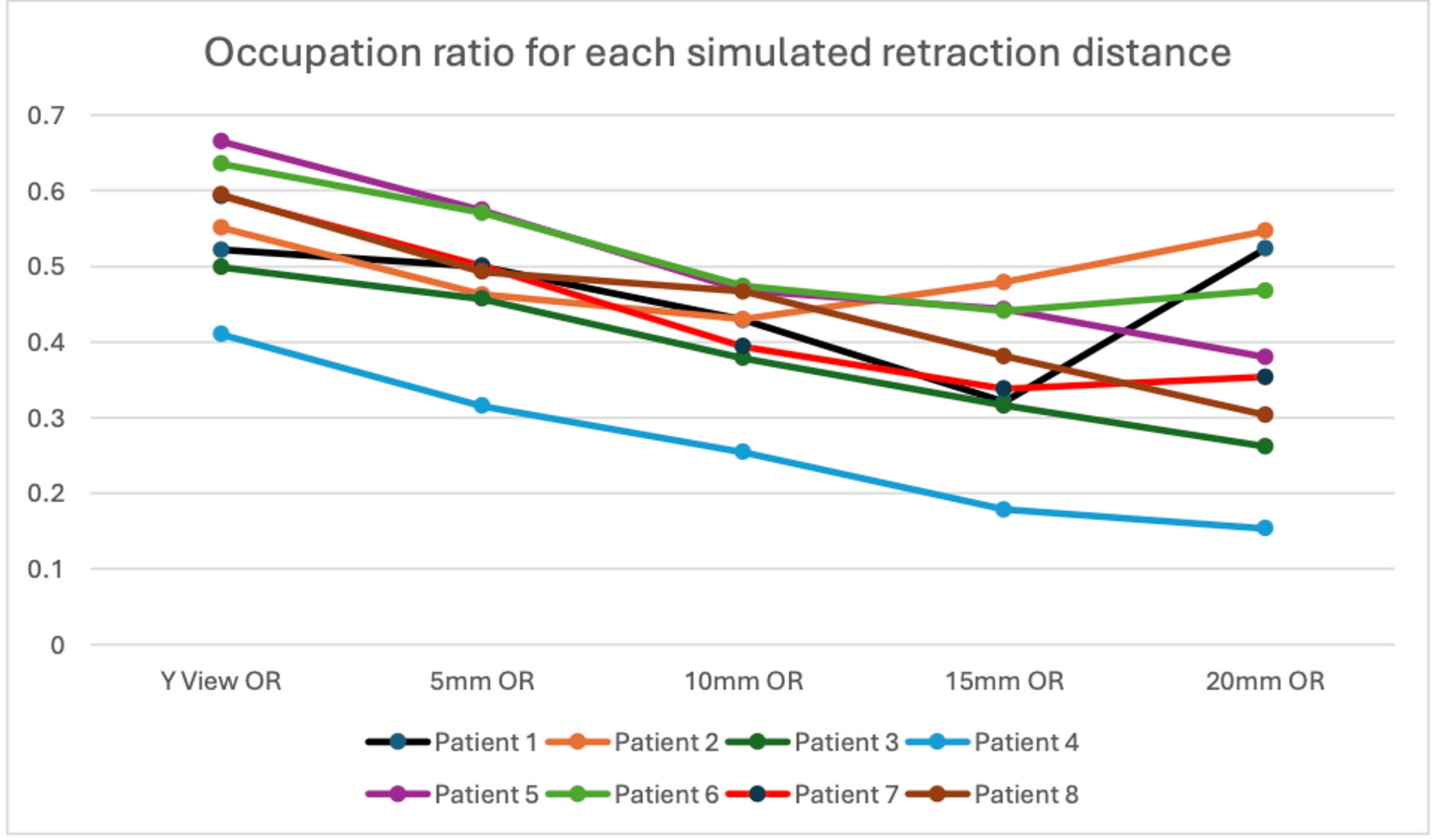
Occupation ratio (OR) by patient at each distance, comparing overall mean values with observer/replicate-specific values.

By using the supraspinatus fossa surface area that was calculated at the Y-view, an occupation ratio was created. A simulated occupation ratio was then created for each distance of simulated retraction. Figure 6 shows that the occupation ratio mirrored the findings from the supraspinatus surface area.

Results from the models confirm this observation. Each 1 mm increase in simulated tear retraction distance was associated with a 30.5 mm² decrease in supraspinatus surface area (95% CI -34.0 to -27.0). However, the model estimating distance-specific means provided a better fit to the data (based on AIC) and indicated that the nadir of supraspinatus surface area occurred at a simulated tear distance of 15 mm (-597.5 mm², 95% CI: -670.6 to -524.3), not 20 mm, suggesting some deviation from linearity. Inter-observer differences were negligible. Table 1 shows the findings of the two models.

**Table 1:** Comparing the two models for supraspinatus surface area at each simulated retraction distance. Model 1: Assumes a linear effect of distance Model 2: Estimates the mean supraspinatus surface area at each distance SSA, supraspinatus surface area; CI, confidence interval

Predictors	SSA (mm^2^)					
	Model 1			Model 2		
	Estimates	CI	P	Estimates	CI	P
Intercept (Distance = 0 mm)	1596.34	1398.23 to 1794.45	<0.001	1661.71	1461.10 to 1862.32	<0.001
Distance (mm)	-30.52	-34.02 to -27.02	<0.001	-	-	-
Distance = 5 mm	-	-	-	-230.01	-303.15 to -156.87	<0.001
Distance = 10 mm	-	-	-	-446.23	-519.37 to -373.09	<0.001
Distance = 15 mm	-	-	-	-597.46	-670.60 to -524.32	<0.001
Distance = 20 mm	-	-	-	-579.39	-652.53 to -506.25	<0.001

When comparing the fit of the two models, Model 2 demonstrated a better fit, as indicated by a lower AIC. A comparison of the random effects in the two models is presented in Table 2.

**Table 2:** Comparison of the random effects from the two models used. σ2: Variance τ00 (patient): Variance associated with the random effects of patients τ00 (observer): Variance associated with the random effects of observers ICC: Intraclass Correlation Coefficient N: Sample size (number of subjects) Observations: Total data points collected Marginal R2: Proportion of variance explained by fixed effects only Conditional R2: Proportion of variance explained by both fixed and random effects AIC: Akaike Information Criterion

Metric	Model 1			Model 2		
σ^2^	37867.45			33070.94		
τ_00_ (patient)	74227.66			74386.13		
τ_00_ (observer)	1079.61			1139.46		
ICC	0.67			0.7		
N (patient)	8			8		
N (Observer)	3			3		
Observations	240			240		
Marginal R^2^/Conditional R^2^	0.418/0.805			0.442/0.830		
AIC	3242.734			3182.469		

**Figure 7:**
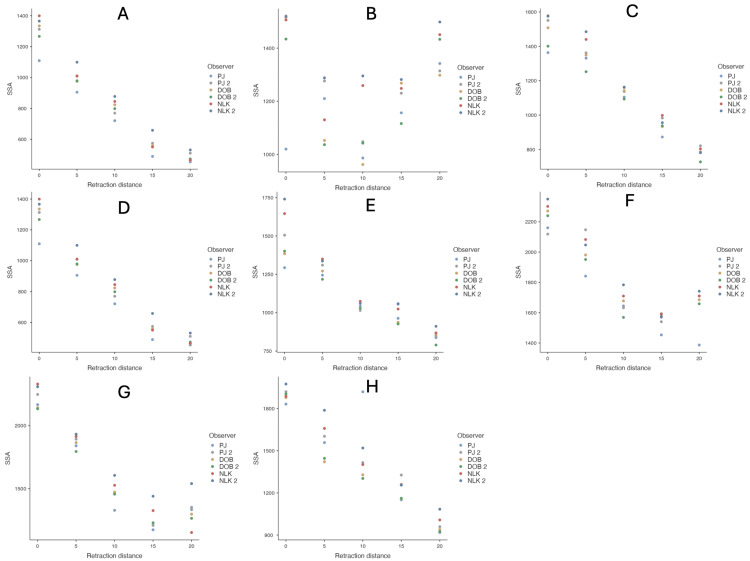
Inter- and intra-rater variability in supraspinatus surface area, with each patient shown in a separate facet. PJ, DOB, and NLK represent the three authors who measured supraspinatus surface area. J2, DOB2, and NLK2 refer to the same authors performing the measurements at a different time interval. (A) to (H) correspond to image planes for Patients 1-8, respectively.

There was good inter- and intra-rater consistency in supraspinatus surface area measurements for each patient, except for Patient 2. This is illustrated in Figure 7, with each patient represented as a separate facet.

## Discussion

Multiple studies have sought to identify clinical and radiological factors that influence rotator cuff reparability [1-6]. A strong association was found between the reparability of the rotator cuff and the following: chronic pseudoparalysis, mediolateral tear size, acromiohumeral distance, tangent sign, fatty infiltration of the muscle (as defined by Goutallier grade), and the extent of the rotator cuff involved in the tear. These factors have been used to create a formula to determine the reparability of the rotator cuff tear [2].

Logit P = 1.264 × Chronic Pseudo Paralysis + 0.084 × Mediolateral tear size - 0.472 × Acromiohumeral Distance + 0.804 × Group 2 Fatty Infiltration of supraspinatus + 1.815 × Tangent sign + 2.514 × Type 4 Tendon Involvement - 3.460

**Figure 8:**
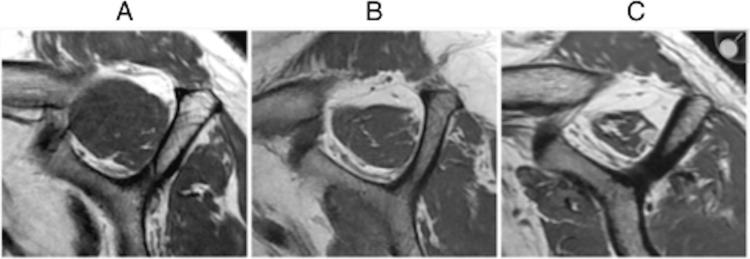
Grading of the occupation ratio as illustrated on MRI scan. (A) Grade 1, minimal to mild atrophy of supraspinatus muscle, occupation ratio ≥60%. (B) Grade 2, moderate atrophy of supraspinatus, occupation ratio 40%-59%. (C) Grade 3, severe atrophy of supraspinatus, occupation ratio <40%. Copyright/license: This figure has been adopted from Lim et al. [22], an open-access article distributed under the terms of the Creative Commons Attribution Non-Commercial License (http://creativecommons.org/licenses/by-nc/3.0/).

Atrophy of the rotator cuff, as indicated by surrogate markers such as the tangent sign, Goutallier grade, and occupation ratio, has been extensively studied as a predictor of reparability [1-3,5,13,14,6,17,11,21]. The occupation ratio is graded from I to III and is illustrated in Figure 8:

Grade I: 0.6-1 was considered normal.

Grade 2: 0.6-0.4 was considered moderate atrophy.

Grade 3: <0.4 indicated severe atrophy.

Although the original evaluation of Goutallier grade was introduced by Goutallier et al. on CT scans in 1994 [7], MRI has become the primary modality for diagnosing supraspinatus muscle atrophy since Fuchs et al. demonstrated a fair to moderate correlation between fatty degeneration of the rotator cuff muscles on CT and MRI in 1999 [8]. The reorientation of images to the parasagittal plane on CT scans by Muller et al. [10] showed comparable outcomes to the original axial views used by Goutallier.

This study used the parasagittal view described by Thomazeau on CT scans to measure the supraspinatus muscle cross-sectional area.

Our study challenged the notion that the occupation ratio in the Y-view can be reliably used in acute rotator cuff tears to determine atrophy and reparability of the rotator cuff. We used basic anatomical principles of the supraspinatus muscle, which is tapering/fusiform in shape, to simulate retraction after a hypothetical acute tear. The distance of retraction was simulated by using the classification of rotator cuff retraction that was described by Patte [12]. We measured up to a simulated tear retraction of 20 mm, as a study by Shah et al. showed an average retraction of 23.0 ± 14.4 mm when radiologically reviewing 25 patients with large rotator cuff tears [13].

All the patients we analyzed had intact rotator cuffs with no fatty infiltration greater than grade 2.

Seven of the eight shoulders demonstrated an increase in their classified stage of atrophy between the 0 mm point and the 20 mm simulated retraction. When using an occupation ratio of less than 0.4 to define severe atrophy, 5 out of 8 patients with simulated non-atrophic retracted tears would have been classified as having severe atrophy at a simulated retraction of 15 mm. This raises concerns for potential decision-making regarding the reparability of this simulated acute tear.

Three studies that further illustrate our findings were conducted by Fukuta et al. [16], Jeong et al. [15], and Jo et al. [17]. Fukuta et al. demonstrated that the cross-sectional area of the supraspinatus muscle in the Y-view cannot be reliably used to assess muscle atrophy. They recommended using more medial images after a tear to better evaluate true muscle mass following acute supraspinatus retraction. Jeong et al. used the Y-view on MRI to measure the cross-sectional area of the supraspinatus muscle following rotator cuff repair and found that the area increased after repair of a retracted supraspinatus. Jo et al. showed improvement in both muscle atrophy grade and the cross-sectional area of the supraspinatus muscle following rotator cuff repair. In both studies assessing postoperative improvement in cross-sectional area and fatty infiltration, MRI scans were performed in the immediate postoperative period, thereby excluding the effects of rehabilitation. Our research suggests that the findings in these studies likely reflect a restoration of pre-tear characteristics, rather than an actual improvement over the premorbid baseline.

Limitations of this study

This study acknowledges certain limitations. The sample size consisted of only eight shoulder CT scans, which may restrict the generalizability of our findings. While this focused analysis provides valuable insights, larger studies are warranted to confirm and build upon our results.

Additionally, variability was observed at the 20 mm simulated retraction, likely due to anatomical overlap between the supraspinatus tendon and the infraspinatus muscle. This overlap may affect measurement accuracy and should be taken into account when interpreting our findings.

Furthermore, it is important to note that ImageJ does not provide metric calibration, which could introduce measurement variability. Future studies may benefit from utilizing calibrated measurement tools to enhance precision in data collection.

Finally, the single-center design of this study may limit the diversity of the patient population, suggesting that multi-center research could enrich our understanding of these dynamics.

## Conclusions

The important clinical relevance of this study is that a simulated acute retracted tear could be incorrectly categorized as an atrophic muscle and could thus influence decision-making about repairability. This reinforces the idea that muscle bulk more medial to the Y-view is more suited to measuring atrophy in the setting of an acute rotator cuff tear.
